# Supplementary material for: Fast and Accurate Prediction of Refractive Index of Organic Liquids with Graph Machines
Source: Molecules. 2023 Sep 26;28(19):6805. doi: 10.3390/molecules28196805 (PMC10574377; doi:10.3390/molecules28196805)
Supplement: Supplementary file 1 [file molecules-28-06805-s001.zip › RIdemo-SI.pdf]

Supporting Information

Fast and Accurate Prediction of Refractive Index of Organic Liquids with Graph Machines

François Duprat [1,*], Jean-Luc Ploix [1], Jean-Marie Aubry [2], Théophile Gaudin [3]

1 Molecular, Macromolecular Chemistry and Materials, ESPCI Paris, PSL Research University, 75005 Paris, France
2 Univ. Lille, CNRS, Centrale Lille, Univ. Artois, UMR 8181 - UCCS – Unité de Catalyse et Chimie du Solide, F-59000 Lille, France
3 Dassault Systemes BIOVIA, CB4 0FJ Cambridge, United Kingdom
* arthur.duprat@espci.psl.eu

A. TEST OF THE “CTn2” MODEL FOR THE REFRACTIVE INDEX

Principle and technical details

BIOVIA COSMOtherm [38-41] ships a prediction method for the refractive index that can readily be used given any COSMO file. The model is hereafter labeled CTn2 and is based on simple atom-parameterized contributions for polarizability α_i for each atom of type i, as well as on the QSPR model predicting molar volume that is also readily shipped within COSMOtherm $V_{m,COSMOtherm}$ (Reference Manual, BIOVIA COSMOtherm 2023, Dassault Systèmes)

$$\frac{n^2-1}{n^2+2} \approx \frac{\sum_i N_i \alpha_i}{3\varepsilon_0 V_{m,COSMOtherm}} \tag{S1}$$

where Ni is the number of atoms of type i (such as C atoms) in the molecule. To date, parameters α_i are unpublished in literature. Note that though this model is part of the COSMOtherm implementation, it is not part of COSMO-RS theory and should be considered a combination of two QSPR models, one for polarizability and one for molar volume.

To obtain the predictions (using the 2023 release of the COSMOtherm), we proceeded as follows: (i) add the keyword “pri1”, designed to print additional output to the .out file from a COSMOtherm calculation, to Global Options. This can be done in the Graphical User Interface by clicking Extras > Global Options and displaying the “Print options for the COSMOtherm output and table files”, (ii) run a “Mixture” job (in the Properties tab) and (iii) extract from the .out file the refractive index that appears in like containing “Molecule <name> N**2 =” with <name> being the name of the molecule (for example, ethanol).

Note that the composition of the mixture defined in the Mixture job is irrelevant as the calculated value of n using this model does not depend on conformer weights. Any composition can be chosen.

Results

For the test of the CTn2 model, compounds of the TCI test set (Section 3.2 of the main text) which are also in the COSMObase 2023 are first retrieved. Indeed, when a compound belongs to the COSMObase 2023, no computational chemistry job is required to first obtain the COSMO files required to trigger the calculation. A prediction computation with the CTn2 model was then performed for the 1174 compounds common to both databases. The results presented in Table S1 and Figure S1 show that COSMOtherm provides a reasonable prediction of the refractive index out-of-the-box if anyone is already using it for its core applications in the area of fluid phase thermodynamics, with a standard error of 0.025 on a range of values spanning about 0.4 unit. This test gives insight of the expected accuracy of a simple atomic contribution model for polarizability.

Table S1. Validation metrics for the CTn2 model on a TCI test set of 1174 compounds.

Metric	RMSE	R^2	MIN [1]	MAX [1]	STE
Value	0.025	0.838	–0.103	0.112	0.017

[1] Minimum and maximum deviations from experiment

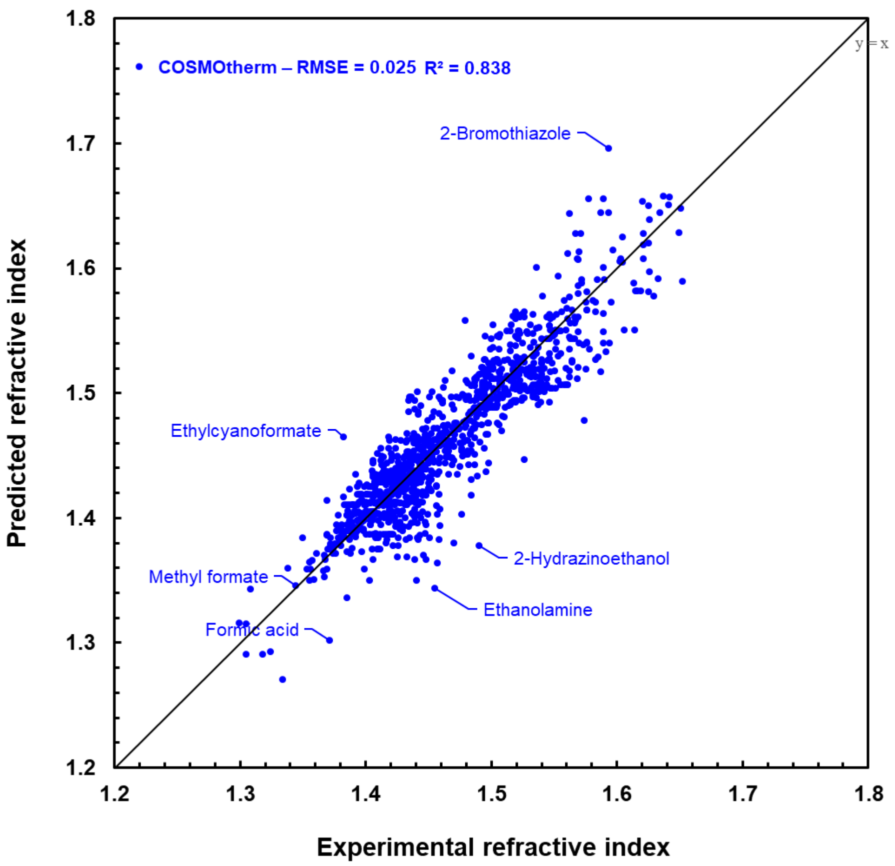

Figure S1. Scatter plot of refractive index predictions computed by COSMOtherm vs measured refractive index values for the 1174 molecules of the TCI test set.

B. DERIVATION OF LORENTZ-LORENZ EQUATION FOR HOMOLOGOUS SERIES

We start with eq. 4 of the main text:

$$\frac{n^2-1}{n^2+2} = k\frac{\alpha}{V} \tag{S2}$$

where we have subsumed $1/3\varepsilon_0$ into a constant k. Rearranging the symbols to express n as a function of other variables yields:

$$n = \sqrt{\frac{2k\frac{\alpha}{V}+1}{1-k\frac{\alpha}{V}}}\ . \tag{S3}$$

We now assume that polarizability and volume are linear functions of the number of a given repeating unit (such as CH_2) in the molecule:

$$\alpha = aN + b \tag{S4}$$

$$V = cN + d \tag{S5}$$

inserting eqs. S4 and S5 in eq. S3 yields:

$$n = \sqrt{\frac{2k\frac{aN+b}{cN+d}+1}{1-k\frac{aN+b}{cN+d}}} \tag{S6}$$

multiplying by cN+d on both sides yields:

$$n = \sqrt{\frac{2k(aN+b)+cN+d}{cN+d-k(aN+b)}} \tag{S7}$$

separating N-dependent terms and constant terms yields:

$$n = \sqrt{\frac{(2ka+c)N+(2kb+d)}{(c-ka)N+(d-kb)}} \tag{S8}$$

dividing by c-ka on both sides yields:

$$n = \sqrt{\frac{\frac{2ka+c}{c-ka}N+\frac{2kb+d}{c-ka}}{N+\frac{d-kb}{c-ka}}} \tag{S9}$$

defining n^2_{repeat} = (2ka+c)/(c-ka), B = (2kb+d)/(c-ka) and C = (d-kb)/(c-ka), we rewrite eq. S9 as:

$$n = \sqrt{\frac{n^2_{repeat}N+B}{N+C}} \tag{S10}$$

which is eq. 9 in the main text.

C. EXPERIMENTAL DATA FOR REFRACTIVE INDICES OF HOMOLOGOUS SERIES

Table S2. Experimental data for refractive indices of homologous series

	series	*n*-alkanes	1-iodoalkanes	1-alkanols	diaminoalkanes	diiodoalkanes	perfluoroalkanes	methylated siloxanes
	initial molecule	ethane	iodomethane	methanol	hydrazine	I_2	perfluoroethane	hexamethyldisiloxane
	repeat unit	CH_2	CH_2	CH_2	CH_2	CH_2	CF_2	$Si(CH_3)_2O$
Number of repeat units	0	n/a	1.531	1.329	1.46044	n/a	n/a	1.378
	1	n/a	1.513	1.361	n/a	1.741	n/a	1.385
	2	n/a	1.506	1.385	1.457	n/a	n/a	1.39
	3	1.358	1.499	1.399	1.458	1.641	1.241	1.393
	4	1.375	1.495	1.41	n/a	1.621	1.252	1.395
	5	1.388	1.492	1.418	1.459	1.603	1.262	1.397
	6	1.398	1.491	1.424	1.459	1.586	1.275	1.397
	7	1.406	1.489	1.43	1.459	1.575	1.281	1.398
	8	1.412	n/a	1.433		1.565		
	9	1.418	1.486	1.437		1.557		1.399
	10	1.422	n/a	1.441				
	11	1.426	1.484	1.441				
	12	1.429						
	13	1.432						
	14	1.435						
	15	1.437	1.48					
	16	1.439						
	17	1.441						
	18	1.442						

D. COSMO-RS ANALYSIS OF TAUTOMERS AND CONFORMERS OF 1,1,1-TRIFLUOROPENTANE-2,4-DIONE

In practice, “conformer sets”, within COSMOtherm, are treated as different microstates of the same compound. As tautomers are different microstates of the same compound at equilibrium, they can be included as part of the same conformer set within COSMOtherm in order to get a self-consistent estimation of the dominant tautomer form in given conditions. With this in mind, we optimized the geometries of 1,1,1-trifluoropentane-2,4-dione as well as the two proposed conformers for the tautomer (*Z*)-5,5,5-trifluoro-4-hydroxypent-3-en-2-one (cf. Figure 4 of the main text) in BIOVIA TURBOMOLE using the job template named “COSMO-BP-TZVPD-FINE”, that is dedicated to produce COSMO files compatible with COSMOtherm calculations at the BP-TZVPD-FINE level. Once the geometry optimizations were completed, we combined the three obtained COSMO files into a so-called “conformer set” within the interface of COSMOtherm. Then, we computed the weights of the different microstates (based on their normalized Boltzmann factors at temperature T = 298.15 K) in the liquid containing only those microstates in equilibrium with each other. The results are listed in Table S3. It can be seen that according to the COSMO-RS calculations, (*Z*)-5,5,5-trifluoro-4-hydroxypent-3-en-2-one with the O-H···O intramolecular H-bond is by far the most stable in this environment.

Table S3. Tautomer and conformer analysis of 1,1,1-trifluoropentane-2,4-dione in its own liquid phase.

Microstate	Relative Gibbs free energy in ideal conductor (ΔG_{COSMO}, kcal.mol^{-1})	COSMO-RS Chemical potential in the liquid phase (μ_{liquid}, kcal.mol^{-1})	Gibbs free energy in the liquid phase (kcal.mol^{-1}) $\Delta G_{liquid} = \Delta G_{COSMO}+\mu_{liquid}$	Microstate weight $\exp(-\Delta G_{liquid,i}/RT)/\Sigma_j \exp(-\Delta G_{liquid,j}/RT)$
1,1,1-trifluoropentane-2,4-dione 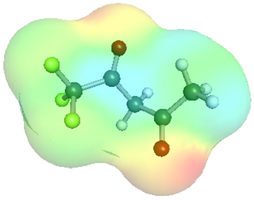	7.17	4.84	12.01	0.0000666%
(Z)-5,5,5-trifluoro-4-hydroxypent-3-en-2-one, O-H···O 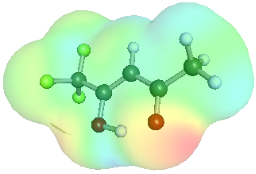	0.00	3.58	3.58	≈100%
(Z)-5,5,5-trifluoro-4-hydroxypent-3-en-2-one, O-H···F 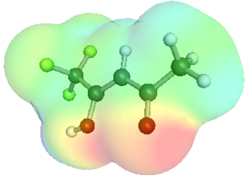	10.69	3.78	14.47	0.00000105%

E. GRAPH MACHINE DEMONSTRATIONS WITH DOCKER CONTAINERS

Installing Docker for Mac and downloading the demo image

In the following example the installation of Docker is performed with an Intel version of Docker Desktop for Mac. The same operations can be done with an Apple ARM-based system. The link to download the ARM version is given at the end of this Section*.

1) Download the Intel chip Docker by clicking the following link:

```
https://desktop.docker.com/mac/main/amd64/Docker.dmg?utm_source=docker&utm_medium=webreferral&utm_campaign=dd-smartbutton&utm_location=module
```

2) After Docker installation (with administrator privileges), launch it. You can open the Docker settings to increase the Docker allocated memory (e.g., 40 Go), and select a number of cores for the virtual machine (more or less according to the machine resources).
3) Open a terminal window, paste the following line, and hit return:

```
docker pull espcigm/metagen-ri
```

The image used to create containers is then downloaded.

4) You can ensure that the image is genuine by checking the hash code generated at the end of the download process; it should be:

```
sha256: fab8cce3976c7890a576421cdb058dc42a8ed4681076755041bcbbac07f409e9
```

The setup is now complete. The demo is safe and ready to use.

More information on Docker client installation can be obtained from the Docker website (www.docker.com).

*The link to install the Apple chip version of Docker is the following:

```
https://desktop.docker.com/mac/main/arm64/Docker.dmg?utm_source=docker&utm_medium=webreferral&utm_campaign=dd-smartbutton&utm_location=module
```

Installing Docker for Windows and downloading the demo image

The steps for the installation of the Docker Windows version and the demo image are given below.

1) Download the Windows version of Docker by clicking the following link:

```
https://desktop.docker.com/win/main/amd64/Docker%20Desktop%20Installer.exe?utm_source=docker&utm_medium=webreferral&utm_campaign=dd-smartbutton&utm_location=module
```

2) Install Docker Desktop from an account with **administrator privileges**
3) Once installation has been successfully completed, open Docker Desktop using the desktop shortcut. You must accept the Docker Subscription Service Agreement by clicking on the 'Accept' box. Continue without signing in, click on skip in the next window, then close the Docker desktop window when the Docker engine has finished starting.
4) Open a PowerShell window (or a command prompt window), paste the following line and hit return:

```
docker pull espcigm/metagen-ri
```

The image used to create containers is then downloaded.

5) You can ensure that the image is genuine by checking the hash code generated at the end of the download process; it should be:

```
sha256: fab8cce3976c7890a576421cdb058dc42a8ed4681076755041bcbbac07f409e9
```

The setup is now complete.

Notes: 1) If Hyper-V is active on your system, Docker can use it instead of WLS 2. To do this, uncheck the 'Use WSL 2 instead of Hyper-V' box in the Docker configuration pop-up window, just before starting the installation.

2) Docker Desktop can be installed for a standard user. The user must be a member of the docker-users group to run Docker. This can be done with the Windows administration tools.

Installing Docker for Ubuntu 20.04 TLS and downloading the demo image

The steps for the installation of the Docker Ubuntu version and the demo image are given below.

1) Open a terminal window, paste the following line, and hit return:

```
sudo snap install docker
```

2) When the installation is complete, download the docker image with the following command:

```
sudo docker pull espcigm/metagen-ri
```

The image used to create containers is then downloaded.

Note: sudo is necessary to connect with the docker daemon which is owned by root. If you don't want to have to preface the docker command with sudo, follow the instructions of this page:
https://docs.docker.com/engine/install/linux-postinstall/

Loading and launching the Docker image

To open a container that will launch the default graph machine computations for the molecules of the *test set*, open a terminal window (or start a PowerShell session), and type the following line of text below (or copy and paste it), the argument demo being optional:

```
docker run -it --rm espcigm/metagen-ri
```

Another computation can be called with the same command line but with the argument demofull. The explanations and the outputs of this command line are given in the Section 'Graph machine results with Docker'.

Notes: 1) No file is created on the host machine; the computed results are lost when the container is deleted.
2) The computed times reported during the demo depend on the machine used.

For more explanations on hyper-V you can use the following link:
https://docs.microsoft.com/fr-fr/virtualization/hyper-v-on-windows/quick-start/enable-hyper-v

Predicting RI for the test set

We explain hereafter the demo that describes the RI computation for the ten test molecules with the selected complexity of the graph machine-based model; the command line for launching this demo is the following:

```
docker run -it --rm espcigm/metagen-ri
```

The data file used for the computation of the RI values of the test set molecules is the file Test175.xlsx, that

can be downloaded separately. The file sheet contains the data for the 175 molecules of the test set. Every line of this sheet contains at least a compound name, its SMILES code and the experimental value of the property of interest (here the refractive index RI). The other data that can be present in the file, e.g. the chemical abstract registry number (CASRN) and the molecular formula (MF), are ignored.

During the training step *achieved in an earlier stage*, a graph machine model based on the SMILES code derived from the molecular structure was automatically generated for every molecule of the training set. All 8267 models were then merged into a module that was trained with the desired property values. The parameters at the end of the training were stored to be used with a new model.

In the course of the present demo a graph machine model is generated for the 10 first molecules (also inputted as their SMILES codes) of the 10 first lines of the sheet. After the model constructions, the parameters saved during the training step are passed to the functions of the 10 graph machines to predict the RI values of the test set molecules.

Explanation of the demo command line

The execution of the graph machine demonstration can be launched from the command line. The proposed default command line is (demo can be omitted):

```
docker run -it --rm espcigm/metagen-ri demo
```

It contains the following terms:

- "docker": calls the Docker daemon of the host machine;
- "run": launches a Docker container from the Docker image;
- "-it": opens and launches the interactive mode;
- "--rm": destroys the container at the end of the session;
- "espcigm/metagen-ri": name of the Docker image launched with the run command;
- "demo": this argument invokes the graph machines mode and a SMILES input is expected. The demo computations are made with the graph machines model that has the number of hidden neurons chosen when looking for the appropriate complexity, i.e. 24 hidden neurons. After completion of the demo, the container is automatically deleted. A new demo session can be started with the same command, but within a new container. If "demofull" is used as argument, instead of "demo", the RI predictions are computed for the 175 compounds of the test set. A progress indicator is then displayed, providing information on the duration of the computation.

Other command line option

One subcommand 'get' can be appended to the command line instead of using demo or demofull. This subcommand, when passed to the demonstrator, allows the computation of the property value for a single compound using a SMILES code input as follows:

```
docker run -it --rm espcigm/metagen-ri get "id;input"
```

where "id" is the name of the compound and "input" corresponds to a SMILES code. When the computation is done, the results are written in the terminal window. Some examples are given in the next Section, paragraph 'Prediction of RI for a single molecule of the test set'.

F. GRAPH MACHINE RESULTS WITH DOCKER

All computations were made on a 3.2 GHz 8-Core Intel Xeon W iMac Pro with 64 GB of RAM running macOS Ventura 13 (Docker configuration: 16 CPUs, 40 GB allocated RAM, 1.5 Go swap). All graph machine tasks were launched with the maximum number of available CPUs minus 2, i.e. 14 CPUs on the iMac Pro.

Predictions of the RI values for the first 10-molecule test set

For the graph machine model with 24 hidden neurons, the predictions for the ten molecules are obtained with the following command (demo can be omitted):

```
docker run -it --rm espcigm/metagen-ri demo
```

The terminal output is then:

```
Run demo computing |
computing time: 22.28 s
results:
+---------------------------+-----------------+-------+----------------+-------------+-------------+
| Name                      | SMILES          |    RI |   Estimated_RI |   minmin_RI |   maxmax_RI |
|---------------------------+-----------------+-------+----------------+-------------+-------------|
| (1Z)-1-Chloro-1-hexene     | CCCC\C=C/Cl     | 1.430 |          1.435 |       1.423 |       1.450 |
| (1E)-1-Chloro-1-hexene     | CCCC/C=C/Cl     | 1.432 |          1.436 |       1.423 |       1.460 |
| (3E)-3-Chloro-3-hexene     | CC/C(Cl)=C/CC   | 1.434 |          1.434 |       1.425 |       1.450 |
| (3Z)-3-Chloro-3-hexene     | CC/C(Cl)=C\CC   | 1.436 |          1.434 |       1.424 |       1.446 |
| (3E)-2,2-Dimethyl-3-hexene | CC(C)(C)/C=C/CC | 1.406 |          1.409 |       1.400 |       1.418 |
| cis-2,2-Dimethyl-3-hexene  | CC(C)(C)/C=C\CC | 1.410 |          1.410 |       1.400 |       1.420 |
| (1Z)-Cyclononene           | C1=C\CCCCCCC/1  | 1.479 |          1.472 |       1.465 |       1.477 |
| (1E)-Cyclononene           | C1=C/CCCCCCC/1  | 1.480 |          1.470 |       1.462 |       1.477 |
| (1E)-Cyclodecene           | C1=C/CCCCCCCC/1 | 1.482 |          1.471 |       1.466 |       1.477 |
| cis-Cyclodecene            | C1=C\CCCCCCCC/1 | 1.485 |          1.473 |       1.467 |       1.477 |
+---------------------------+-----------------+-------+----------------+-------------+-------------+
```

In this case, the RI predictions are the same than those reported as prediction values in column G of the Table S14 for the first 10 test molecules (entries 1-10).

Estimations of the RI values for the 175-molecule test set

For the graph machine model with 24 hidden neurons, the estimations for the 175 molecules of the test set are obtained with the following command:

```
docker run -it --rm espcigm/metagen-ri demofull
```

The terminal output is then:

```
Run demo full test
computing /
computing time: 05:53.63
results:
```

Name	SMILES	RI	Estimated_RI	minmin_RI	maxmax_RI
(1Z)-1-Chloro-1-hexene	CCCC\C=C/Cl	1.430	1.435	1.423	1.450
(1E)-1-Chloro-1-hexene	CCCC/C=C/Cl	1.432	1.436	1.423	1.460
(3E)-3-Chloro-3-hexene	CC/C(Cl)=C/CC	1.434	1.434	1.425	1.450
(3Z)-3-Chloro-3-hexene	CC/C(Cl)=C\CC	1.436	1.434	1.424	1.446
(3E)-2,2-Dimethyl-3-hexene	CC(C)(C)/C=C/CC	1.406	1.409	1.400	1.418
cis-2,2-Dimethyl-3-hexene	CC(C)(C)/C=C\CC	1.410	1.410	1.400	1.420
(1Z)-Cyclononene	C1=C\CCCCCCC/1	1.479	1.472	1.465	1.477
(1E)-Cyclononene	C1=C/CCCCCCC/1	1.480	1.470	1.462	1.477
(1E)-Cyclodecene	C1=C/CCCCCCCC/1	1.482	1.471	1.466	1.477
cis-Cyclodecene	C1=C\CCCCCCCC/1	1.485	1.473	1.467	1.477
(2Z)-2,4-Pentadienenitrile	C=C\C=C/C#N	1.486	1.491	1.470	1.511
(2E)-2,4-Pentadienenitrile	C=C/C=C/C#N	1.499	1.493	1.474	1.516
cis-Cinnamyl acetate	CC(OC/C=C\C1=CC=CC=C1)=O	1.525	1.542	1.530	1.551
trans-Cinnamyl acetate	CC(OC/C=C/C1=CC=CC=C1)=O	1.544	1.545	1.536	1.554
cis-beta-Methylstyrene	C/C=C\C1=CC=CC=C1	1.543	1.548	1.537	1.555
trans-beta-Methylstyrene	C/C=C/C1=CC=CC=C1	1.550	1.554	1.544	1.562
cis-Cinnamyl alcohol	OC/C=C\C1=CC=CC=C1	1.570	1.574	1.562	1.587
trans-Cinnamyl alcohol	OC/C=C/C1=CC=CC=C1	1.582	1.581	1.568	1.592
cis-alpha-Ocimene	C=C/C(C)=C\CCC(C)=C	1.479	1.478	1.468	1.489
trans-alpha-Ocimene	C=C/C(C)=C/CCC(C)=C	1.480	1.478	1.469	1.490
(1alpha,2alpha,3alpha)-1,2,3-Trimethylcyclopentane	C[C@@H]1C(C)[C@@H](C)CC1	1.422	1.428	1.419	1.438
(1alpha,2alpha,3alpha)-1,2,3-Trimethylcyclopentane	C[C@@H]1[C@H](C)[C@H](C)CC1	1.425	1.428	1.419	1.438
rel-(2R,3R)-3-Chloro-2-butanol	C[C@@H](O)[C@H](Cl)C	1.439	1.438	1.431	1.446
rel-(2R,3S)-3-Chloro-2-butanol	C[C@H](O)[C@H](Cl)C	1.440	1.440	1.428	1.449
cis-3-Methylcyclopentanol	O[C@H]1C[C@@H](C)CC1	1.446	1.448	1.440	1.457
trans-3-Methylcyclopentanol	O[C@H]1C[C@H](C)CC1	1.446	1.450	1.438	1.463
cis-Octahydropentalene	[C@H]12CCC[C@H]1CCC2	1.462	1.459	1.453	1.464
trans-Octahydropentalene	[C@H]12CCC[C@@H]1CCC2	1.462	1.460	1.448	1.472
rel-(2R,3S)-3-Bromo-2-butanol	C[C@H](O)[C@H](Br)C	1.476	1.475	1.463	1.485

rel-(2R,3R)-3-Bromo-2-butanol	C[C@@H](O)[C@H](Br)C	1.477	1.474	1.466	1.481
rel-(1R,2R)-2-(2-Propen-1-yl)cyclohexanol	O[C@H]1[C@@H](CC=C)CCCC1	1.476	1.481	1.474	1.487
rel-(1R,2S)-2-(2-Propenyl)cyclohexanol	O[C@H]1[C@H](CC=C)CCCC1	1.478	1.482	1.474	1.486
rel-(1R,2S)-2-Chlorocyclohexanol	O[C@H]1[C@@H](Cl)CCCC1	1.489	1.488	1.480	1.496
rel-(1R,2R)-2-Chlorocyclohexanol	O[C@H]1[C@H](Cl)CCCC1	1.491	1.489	1.482	1.496
(+)-trans-Carveol	CC1=CC[C@H](C(C)=C)C[C@H]1O	1.496	1.503	1.488	1.522
(+)-cis-Carveol	CC1=CC[C@H](C(C)=C)C[C@@H]1O	1.496	1.502	1.486	1.516
rel-(2R,3S)-2,3-Dibromobutane	C[C@@H](Br)[C@@H](Br)C	1.511	1.512	1.503	1.523
DL-2,3-Dibromobutane	C[C@@H](Br)[C@H](Br)C	1.515	1.513	1.505	1.522
(E)-1-bromo-2-fluoroethene	F/C=C/Br	1.406	1.414	1.393	1.444
(4Z)-4-Chloro-4-octene	CCC/C(Cl)=C\CCC	1.442	1.442	1.433	1.452
(2Z)-4-Methyl-2-pentenoic acid	CC(C)/C=C\C(O)=O	1.445	1.441	1.431	1.452
(2E)-1-Chloro-2-methyl-2-butene	C/C=C(C)/CCl	1.448	1.444	1.439	1.451
trans-2-Butene-1,4-diol	C(/C=C/CO)O	1.476	1.479	1.471	1.488
Ethyl (E,Z)-2,4-decadienoate	CCCCC\C=C/C=C/C(OCC)=O	1.488	1.483	1.471	1.503
(3E,5E)-1,3,5-Heptatriene	C=C/C=C/C=C/C	1.528	1.520	1.510	1.532
rel-(2R,5R)-2,5-Dimethylcyclohexanone	N#C/C=C/C1=CC=CC=C1	1.603	1.593	1.579	1.604
(2E)-3-Phenyl-2-propenenitrile	O=C1[C@H](C)CC[C@@H](C)C1	1.444	1.444	1.436	1.454
(+)-Dibutyl tartrate	CCCCOC(=O)[C@@H]([C@H](C(=O)OCCCC)O)O	1.445	1.451	1.440	1.464
meso-3,4-Dibromohexane	CC[C@H](Br)[C@H](Br)CC	1.508	1.509	1.499	1.519
Methyl abietate	CC(C)C1=CC2=CC[C@@H]3[C@@]([C@H]2CC1)(CCC[C@@]3(C)C(=O)OC)C	1.534	1.526	1.512	1.540
Perfluorononane	FC(F)(F)C(F)(F)C(F)(F)C(F)(F)C(F)(F)C(F)(F)C(F)(F)C(F)(F)C(F)(F)F	1.280	1.269	1.260	1.279
1,2-Dichloro-1,1,3,3,3-pentafluoropropane	C(C(F)(F)F)(C(F)(F)Cl)Cl	1.323	1.322	1.310	1.334
3-Chloro-1,1,2,2-tetrafluoropropane	C(C(C(F)F)(F)F)Cl	1.326	1.329	1.322	1.335
Dimethyl fluorophosphate	O=P(OC)(F)OC	1.354	1.360	1.346	1.372
1-Fluoropentane	CCCCCF	1.359	1.360	1.353	1.368
Ethane, isocyano-	CC[N+]#[C-]	1.362	1.366	1.344	1.393
2,3-Dichloro-1,1,1-trifluoropropane	C(C(C(F)(F)F)Cl)Cl	1.367	1.370	1.361	1.378
Methyl isobutyl ether	COCC(C)C	1.369	1.369	1.365	1.373
tert-Butanol	CC(C)(C)O	1.384	1.384	1.377	1.388
Diethylsilane	CC[SiH2]CC	1.392	1.371	1.316	1.405
Triethylborane	B(CC)(CC)CC	1.397	1.398	1.379	1.417

2-Methyl-2-nitropropane	CC(C)(C)[N+](=O)[O-]	1.402	1.401	1.391	1.410
1-(Ethynyloxy)butane	CCCCOC#C	1.402	1.403	1.393	1.414
3-Methyl-2-butyl acetate	CC(OC(C)C(C)C)=O	1.402	1.401	1.395	1.407
tert-Butyl hypochlorite	CC(C)(C)OCl	1.403	1.396	1.373	1.416
Tetradecamethylcycloheptasiloxane	C[Si]1(C)O[Si](C)(C)O[Si](C)(C)O[Si](C)(C)O[Si](C)(C)O[Si](C)(C)O[Si](C)(C)O1	1.404	1.399	1.390	1.411
2,2,4-Trimethyl-3-pentanone	CC(C)C(=O)C(C)(C)C	1.406	1.412	1.407	1.417
Tetra-sec-butoxysilane	CCC(O[Si](OC(C)CC)(OC(C)CC)OC(C)CC)C	1.408	1.402	1.385	1.422
Tetrachloro-1,2-difluoroethane	C(C(F)(Cl)Cl)(F)(Cl)Cl	1.413	1.411	1.407	1.417
3,3-Dimethyl-2-butanol	CC(C)(C)C(C)O	1.415	1.414	1.409	1.419
Diethyl isopropylphosphonate	CC(P(OCC)(OCC)=O)C	1.415	1.422	1.414	1.430
Propylene carbonate	O=C1OC(C)CO1	1.421	1.418	1.410	1.425
2-Methyl-4-heptanol	CCCC(CC(C)C)O	1.422	1.423	1.418	1.426
Ethyl P,P-dimethylphosphinate	O=P(C)(C)OCC	1.429	1.424	1.413	1.443
2-Tridecanone	CCCCCCCCCCCC(=O)C	1.432	1.436	1.433	1.439
2-Dodecanone	CCCCCCCCCCC(=O)C	1.433	1.433	1.430	1.436
Chloroacetone	CC(=O)CCl	1.434	1.428	1.424	1.432
Dimethyl P-octylphosphonate	CCCCCCCCP(OC)(OC)=O	1.435	1.438	1.430	1.446
Heptadecane	CCCCCCCCCCCCCCCCC	1.437	1.434	1.432	1.436
5-Undecyne	CCCCC#CCCCCC	1.437	1.437	1.432	1.443
Dodecyldimethylsilane	C[Si](CCCCCC)(CCCCCC)C	1.437	1.441	1.431	1.448
Butylbis(1,1-dimethylethyl)borane	CC(B(CCCC)C(C)(C)C)(C)C	1.437	1.440	1.424	1.455
4-Undecyne	CCCC#CCCCCCC	1.438	1.438	1.432	1.442
Tetramethyltin	C[Sn](C)(C)C	1.441	1.461	1.434	1.498
Dodecylamine	CCCCCCCCCCCCN	1.442	1.444	1.442	1.446
Methyl cyanoacrylate	COC(=O)C(=C)C#N	1.443	1.446	1.436	1.455
Undecylenic acid	C=CCCCCCCCCC(=O)O	1.449	1.450	1.446	1.452
1-Chlorooctadecane	CCCCCCCCCCCCCCCCCCCl	1.452	1.451	1.449	1.453
Dibutyldiethylgermane	CC[Ge](CCCC)(CCCC)CC	1.452	1.456	1.448	1.463
Cyclohexanecarboxylic acid	C1CCC(CC1)C(=O)O	1.453	1.464	1.460	1.470
6-Undecynoic acid	CCCCC#CCCCCC(O)=O	1.457	1.461	1.456	1.467
2-Chlorofuran	ClC1=CC=CO1	1.457	1.469	1.453	1.482
Tetradecylcyclohexane	CCCCCCCCCCCCCCC1CCCCC1	1.458	1.457	1.454	1.460

Triethylhexylgermane	CC(C)CC[Ge](CCC(C)C)(CCC(C)C)CCC(C)C	1.458	1.456	1.441	1.469
1-Methylcyclohexanol	CC1(CCCCC1)O	1.460	1.459	1.454	1.462
3-Chlorofuran	ClC1=COC=C1	1.460	1.477	1.462	1.487
(±)-Linalool	CC(=CCCC(C)(C=C)O)C	1.461	1.461	1.457	1.465
1-Bromooctadecane	CCCCCCCCCCCCCCCCCCBr	1.463	1.462	1.460	1.465
Cyclohexanol	C1CCC(CC1)O	1.464	1.461	1.458	1.464
1-Octadecanethiol	CCCCCCCCCCCCCCCCCCS	1.465	1.465	1.462	1.468
1,1-Sulfinylbis[propane]	CCCS(=O)CCC	1.466	1.463	1.450	1.472
1,3-Glyceryl dinitrate	C(C(CO[N+](=O)[O-])O)O[N+](=O)[O-]	1.471	1.457	1.380	1.521
delta-Coniceine	C1CCN2CCCC2C1	1.471	1.473	1.469	1.478
2-Furanacetonitrile	N#CCC1=CC=CO1	1.471	1.476	1.469	1.483
Tetraethyltin	CC[Sn](CC)(CC)CC	1.473	1.484	1.471	1.505
Nitroglycerin	C(C(CO[N+](=O)[O-])O[N+](=O)[O-])O[N+](=O)[O-]	1.473	1.456	1.364	1.531
9-Undecynoic acid, methyl ester	COC(CCCCCCCC#CC)=O	1.473	1.451	1.446	1.455
4-Morpholineethanamine	C1COCCN1CCN	1.474	1.478	1.473	1.483
1-Ethynylcyclopentanol	C#CC1(CCCC1)O	1.475	1.472	1.461	1.483
Tetrapropylstannane	CCC[Sn](CCC)(CCC)CCC	1.475	1.483	1.472	1.496
1-(3-Furyl)-4-methyl-1-pentanone	O=C(C1=COC=C1)CCC(C)C	1.478	1.492	1.479	1.498
Diethyl selenide	CC[Se]CC	1.479	1.486	1.473	1.494
Hexadecylbenzene	CCCCCCCCCCCCCCCCC1=CC=CC=C1	1.481	1.479	1.476	1.484
Sulfolane	C1CCS(=O)(=O)C1	1.483	1.493	1.482	1.501
Allyltributyltin	C=CC[Sn](CCCC)(CCCC)CCCC	1.483	1.493	1.486	1.501
N-Formylmorpholine	C1COCCN1C=O	1.485	1.487	1.479	1.496
Triethanolamine	C(CO)N(CCO)CCO	1.485	1.496	1.487	1.501
Ethyl 4-fluorobenzoate	CCOC(=O)C1=CC=C(C=C1)F	1.486	1.486	1.482	1.490
Tributyltin oxide	CCCC[Sn](CCCC)(CCCC)O[Sn](CCCC)(CCCC)CCCC	1.487	1.494	1.443	1.609
[1,1-Bicyclopentyl]-2-ol	C1CCC(C1)C2CCCC2O	1.489	1.495	1.489	1.501
1,10-Dibromodecane	C(CCCCCBr)CCCCBr	1.493	1.495	1.491	1.498
Tripropyltin chloride	CCC[Sn](CCC)(CCC)Cl	1.496	1.493	1.482	1.506
Pyrimidine	C1=CN=CN=C1	1.496	1.518	1.498	1.538
2,4-Hexadien-1-ol	CC=CC=CCO	1.498	1.492	1.479	1.503
Phorone	CC(=CC(=O)C=C(C)C)C	1.500	1.491	1.474	1.508

2-Acetylfuran	CC(=O)C1=CC=CO1	1.502	1.492	1.484	1.501
Hexanophenone	CCCCCC(=O)C1=CC=CC=C1	1.503	1.511	1.508	1.514
Methylphenylsilane	C[SiH2]C1=CC=CC=C1	1.506	1.498	1.434	1.536
4-Fluorobenzyl alcohol	C1=CC(=CC=C1CO)F	1.508	1.511	1.507	1.516
2-Methyl-4-phenyl-2-butanol	CC(C)(CCC1=CC=CC=C1)O	1.508	1.508	1.503	1.516
Butylacetanilide	CCCCN(C1=CC=CC=C1)C(=O)C	1.515	1.530	1.522	1.537
Dicyclohexylphosphine	C1CCC(CC1)PC2CCCCC2	1.516	1.518	1.504	1.535
2-Methyl-1-phenyl-2-propanol	CC(C)(CC1=CC=CC=C1)O	1.517	1.511	1.505	1.518
4-tert-Butylacetophenone	CC(=O)C1=CC=C(C=C1)C(C)(C)C	1.518	1.518	1.512	1.524
2-Fluoro-1-phenylethanone	C1=CC=C(C=C1)C(=O)CF	1.520	1.522	1.514	1.536
2-tert-Butyl-6-methylphenol	CC1=C(C(=CC=C1)C(C)(C)C)O	1.520	1.520	1.516	1.524
Chlormephos	CCOP(=S)(OCC)SCCl	1.520	1.520	1.480	1.557
Ethyl 4-phenyl-1-[2-[(tetrahydro-2-furanyl)methoxy]ethyl]-4-piperidinecarboxylate	CCOC(=O)C1(CCN(CC1)CCOCC2CCCO2)C3=CC=CC=C3	1.522	1.517	1.499	1.531
2-Cyanopyridine	C1=CC=NC(=C1)C#N	1.524	1.531	1.524	1.539
3-Isopropylphenol	CC(C)C1=CC(=CC=C1)O	1.526	1.522	1.518	1.528
Dill apiole	COC1=C(C2=C(C=C1CC=C)OCO2)OC	1.529	1.550	1.536	1.562
alpha-Methyl-N-(2,2,2-trichloroethylidene)benzeneethanamine	CC(CC1=CC=CC=C1)N=CC(Cl)(Cl)Cl	1.530	1.544	1.528	1.562
4-Fluoronitrobenzene	C1=CC(=CC=C1[N+](=O)[O-])F	1.532	1.526	1.517	1.533
Benzoic acid, 2-hydroxy-3-methyl-, methyl ester	CC1=C(C(=CC=C1)C(=O)OC)O	1.535	1.538	1.532	1.543
Apiole	COC1=C2C(=C(C(=C1)CC=C)OC)OCO2	1.536	1.542	1.525	1.565
Benzyl azide	C1=CC=C(C=C1)CN=[N+]=[N-]	1.537	1.537	1.511	1.580
Pheniprazine	NNC(C)CC=1C=CC=CC1	1.539	1.533	1.501	1.546
Guaiacol	COC1=CC=CC=C1O	1.543	1.542	1.537	1.546
Tris(ethylthio)borane	CCSB(SCC)SCC	1.547	1.543	1.519	1.589
4-Bromotoluene	CC1=CC=C(C=C1)Br	1.548	1.555	1.549	1.561
Phenelzine	NNCCC1=CC=CC=C1	1.549	1.546	1.519	1.564
alpha-Ethynylbenzenemethanol	C#CC(C1=CC=CC=C1)O	1.551	1.549	1.535	1.560
(±)-Pheniramine	CN(CCC(C1=CC=CC=N1)C2=CC=CC=C2)C	1.552	1.556	1.546	1.566
Dihydrocoumarin	C1CC(=O)OC2=CC=CC=C21	1.556	1.546	1.536	1.553
Tri-m-cresyl phosphate	CC1=CC(=CC=C1)OP(=O)(OC2=CC=CC(=C2)C)OC3=CC=CC(=C3)C	1.557	1.557	1.534	1.574
3,4,5,6-Tetrahydro-5,5-dimethyl-1H-germolo[3,4-c]thiophene	C[Ge](C1)(C)CC2=C1CSC2	1.560	1.544	1.504	1.579
Benzathine	C1=CC=C(C=C1)CNCCNCC2=CC=CC=C2	1.564	1.575	1.557	1.596

2-Iodofuran	IC1=CC=CO1	1.567	1.583	1.561	1.604
Dinitrofluorobenzene	C1=CC(=C(C=C1[N+](=O)[O-])[N+](=O)[O-])F	1.569	1.574	1.541	1.618
Diphenylmethane	C1=CC=C(C=C1)CC2=CC=CC=C2	1.575	1.576	1.570	1.583
4-Methoxybenzoyl chloride	COC1=CC=C(C=C1)C(=O)Cl	1.580	1.567	1.559	1.579
Diphenylsilane	C1([SiH2]C2=CC=CC=C2)=CC=CC=C1	1.580	1.564	1.511	1.591
Phenylphosphine	C1=CC=C(C=C1)P	1.580	1.588	1.515	1.646
Diphenyl ether	C1=CC=C(C=C1)OC2=CC=CC=C2	1.581	1.580	1.571	1.591
Methyl anthranilate	COC(=O)C1=CC=CC=C1N	1.582	1.569	1.562	1.577
o-Chlorobenzotrichloride	C1=CC=C(C(=C1)C(Cl)(Cl)Cl)Cl	1.584	1.571	1.564	1.579
2,3-Dimethyl-5-phenylfuran	CC1=C(C)OC(C2=CC=CC=C2)=C1	1.587	1.578	1.560	1.595
Triphenyl phosphite	C1=CC=C(C=C1)OP(OC2=CC=CC=C2)OC3=CC=CC=C3	1.590	1.586	1.562	1.602
2-Bromobenzaldehyde	C1=CC=C(C(=C1)C=O)Br	1.593	1.597	1.590	1.603
1-Chloro-4-[(chloromethyl)thio]benzene	C1=CC(=CC=C1SCCl)Cl	1.606	1.612	1.605	1.624
Isoquinoline	C1=CC=C2C=NC=CC2=C1	1.615	1.620	1.611	1.634
Benzyl iodide	C1=CC=C(C=C1)CI	1.633	1.628	1.618	1.638
Thiochromanone	C1CSC2=CC=CC=C2C1=O	1.640	1.635	1.610	1.657
1,1-Selenobis[benzene]	C1([Se]C2=CC=CC=C2)=CC=CC=C1	1.649	1.652	1.627	1.668
1,2-Diiodobenzene	C1=CC=C(C(=C1)I)I	1.718	1.725	1.708	1.739

ne

Prediction of RI for a single molecule of the test set

RI prediction of a given test molecule can be replicated for the default graph-machine-based model. Isocyanoethane is given as an example. According to Table S14 in the RI8267A-175T-EXO-SI.xlsx file, the following information can be extracted (entry 56) for this molecule:

Compound	SMILES code	RI_{exp}
isocyanoethane	CC[N+]#[C-]	1.362

The command used to predict its RI from SMILES is the following:

```
docker run -it --rm espcigm/metagen-ri get "isocyanoethane;CC[N+]#[C-]"
```

The output produced is then:

```
computing time: 5.09 s
results:
+----------------+-------------+----------------+-------------+-------------+
| ID             | smiles      |   Estimated_RI |   minmin_RI |   maxmax_RI |
|----------------+-------------+----------------+-------------+-------------|
| isocyanoethane | CC[N+]#[C-] |          1.366 |       1.344 |       1.393 |
+----------------+-------------+----------------+-------------+-------------+
done
```

The results are identical to those computed for isocyanoethane in column G of the S14 table.

Prediction of RI for other compounds

RI Prediction for any liquid of molecule containing atoms among the sixteen atoms present in the training data set, namely carbon, hydrogen, oxygen, nitrogen, halogens, boron, sulfur, phosphorus, silicon, tin, germanium, selenium and titanium, can also be computed with a similar command line starting from its SMILES code.

Phosphinic acid that belongs to the exotic set is used as an example. According to Table S15 in the RI8267A-175T-EXO-SI.xlsx file, the following information can be extracted (entry 14):

Compound	SMILES code	RI_{exp}
Phosphinic acid	O=P([H])([H])O	1.457

The command below is then entered in the terminal window to predict the RI value of phosphinic acid from the SMILES input:

```
docker run -it --rm espcigm/metagen-ri get "phosphinic acid;O=P([H])([H])O"
```

The messages returned are the following:

```
computing time: 5.38 s
results:
+----------------------+----------------+----------------+-------------+-------------+
| ID                   | smiles         |   Estimated_RI |   minmin_RI |   maxmax_RI |
|----------------------+----------------+----------------+-------------+-------------|
| phosphinic acid      | O=P([H])([H])O |          1.460 |       1.219 |       1.721 |
+----------------------+----------------+----------------+-------------+-------------+
done
```

The RI prediction is equal to 1.460 for the GM method, which is also very close to the experimental value given in Table S15 (1.457).

It should however be noted that the predicted confidence interval for the refractive index of phosphinic acid is very large (1.721-1.219=0.502!), whereas it is of the order of 0.026 on average for the 175 molecules in the test set (see previous table issued with argument 'demofull'). This indicates that the value predicted by GM24 for this

compound, even if it approaches the experimental value, should be taken with great caution, as this structure is probably very poorly represented in the training set of 8267 compounds.
